# Building Authentic Connection in the Patient-Physician Relationship

**DOI:** 10.1177/21501319231225996

**Published:** 2024-01-28

**Authors:** Sheryl Fuehrer, Amy Weil, Lars G. Osterberg, Donna M. Zulman, Matthew R. Meunier, Rachel Schwartz

**Affiliations:** 1 Mayo Clinic, Rochester, MN, USA; 2 University of North Carolina at Chapel Hill, Chapel Hill, NC, USA; 3 Stanford University School of Medicine, Palo Alto, CA, USA; 4 VA Palo Alto Health Care System, Palo Alto, CA, USA; 5 VA Palo Alto Health Care System, Menlo Park, CA, USA; 6 University of California, San Francisco, CA, USA

**Keywords:** physician-patient communication, primary care, qualitative research, relationship-centered care, medical education

## Abstract

**Introduction/Objectives:**

Delivering optimal patient care is impacted by a physician’s ability to build trusting relationships with patients. Identifying techniques for rapport building is important for promoting patient-physician collaboration and improved patient outcomes. This study sought to characterize the approaches highly skilled primary care physicians (PCPs) use to effectively connect with diverse patients.

**Methods:**

Using an inductive thematic analysis approach, we analyzed semi-structured interview transcripts with 10 PCPs identified by leadership and/or colleagues for having exceptional patient communication skills. PCPs practiced in 3 diverse clinic settings: (1) academic medical center, (2) Veterans Affairs clinic, and (3) safety-net community clinic.

**Results and Conclusions:**

The thematic analysis yielded 5 themes that enable physicians to establish connections with patients: Respect for the Patient, Engaged Curiosity, Focused Listening, Mutual Participation, and Self-Awareness. Underlying all of these themes was a quality of authenticity, or a state of symmetry between one’s internal experience and external words and actions. Adopting these communication techniques while allowing for adaptability in order to remain authentic in one’s interactions with patients may facilitate improved connection and trust with patients. Encouraging physician authenticity in the patient-physician relationship supports a shift toward relationship-centered care. Additional medical education training is needed to facilitate authentic connection between physicians and patients.

## Introduction

The importance of the patient-physician relationship has been noted since the time of Hippocrates, with the progenitors of medicine highlighting that without this relationship, it is impossible to care adequately for the patient.^1^ The importance of the clinical relationship is clearly evident through studies that have examined the effect of clinical rapport on the Quadruple Aims of improving patient experience, provider experience, and population health outcomes, and reducing healthcare costs.^2-4^ Additional data reveal that a strong patient-physician relationship is linked to increased patient adherence to medical recommendations, symptom improvements, and reduced medical errors.^5,6^

Multiple models and frameworks have been created to provide physicians with communication and behavioral techniques that foster rapport building, connection, and trust–all of which strengthen the patient-physician relationship. Some examples include the Four Habits Model, which was developed to improve physician and patient communication by suggesting 4 practices, or “habits,” to build rapport, and facilitate the effective exchange of information, demonstrate care and concern, and increase the likelihood of positive health outcomes.^7^ Additional communication frameworks include PEARLS, a mnemonic to help providers remember empathic expressions as useful tools for relationship building,^8^ and the Presence 5,^9^ a set of evidence-based, Delphi panel, and observation-based practices to foster physician presence and clinical rapport. While the intention of these frameworks is to advance physician ability to successfully navigate patient encounters, only the more recent frameworks have begun placing an emphasis on physician-internal experiences rather than focusing on what language to use to externally portray “empathy.”

Relationship-Centered Care (RCC) is a care delivery approach in which an emphasis is placed on the relationship between individuals in the clinical encounter, rather than the exchange being transactional or service-oriented. The pillars of RCC include learning to acknowledge the personhood of participants, acknowledging emotion as part of the relationship, understanding that the relationship is reciprocal, and placing an emphasis on the value of it being a genuine relationship.^10^

While the use of RCC physician-patient communication frameworks has been linked to increased patient satisfaction and clinician well-being,^11^ many physicians anecdotally report that they fear a prescriptive approach to communication will come across as disingenuous if delivered without an authentic accompanying sentiment. As medicine becomes an increasingly customer-service oriented endeavor, the bi-directional, reciprocal quality of the healing relationship may be overlooked; however, given the high rates of physician burnout,^12^ and evidence that meaningful, bi-directional professional collaborative relationships support well-being,^13^ new strategies that support clinical rapport are needed.

This study sought to identify how expert physicians build connections with patients, including, but not limited to, communication and behavioral techniques.

## Methods

This work was part of a 3-year study on fostering patient-physician connection in the primary care setting.^9^ Semi-structured interviews with 10 exemplary primary care physicians were conducted by researchers as part of a previous study (see Brown-Johnson et al^14^ for additional details and Appendix A for interview guide). The 10 primary care physicians practiced in 3 diverse clinic settings (an academic medical center, a US Federally Qualified Health Center serving primarily Spanish-speaking immigrants, and a Veterans Affairs facility). The goal of these interviews was to learn about what clinician behaviors and communication techniques best facilitate interpersonal connection and rapport with patients. Since the aim was to identify positive outliers, participating physicians were selected based on recommendations by clinic leadership and physician peers as having exceptional interpersonal skills with patients. The semi-structured interviews were approximately 30 min long and explored how providers build connection and trust with their patients.

De-identified transcripts of these interviews were coded by the first and senior authors to identify core themes. The 2 coders coded the transcripts both individually and together to conduct an inter-coder reliability check and align on coding practices. All discrepancies were resolved by consensus. The research team maintained reflexivity by examining and discussing assumptions throughout the analytic process. This included an awareness of our respective demographics and biases, and, at the time, our non-clinical training in healthcare. The first author had a master’s degree in community health prevention research at the time of data analysis, and is currently completing her medical training. The senior author is a health services researcher and communication scientist with qualitative research expertise. Using an inductive thematic analysis approach to identify latent themes,^15^ we identified physician approaches to connecting with their patients. Initial themes were then discussed and iteratively refined with a third coder and co-author, LGO, an internal medicine physician and medical educator, to arrive at the final themes.

## Results

Interviews were conducted with 10 internal medicine physicians (5 Female; 5 Male), White (30%), Asian (30%), South Asian (20%), Latino (10%), and Black (10%). What emerged from the thematic analysis were 5 themes for establishing connection with patients: Respect for the patient, Engaged Curiosity, Focused Listening, Mutual Participation, and Self-Awareness.

### *Respect for the Patient*: Appreciation for the Patient as a Whole Person, Including Their Beliefs, Values, Personal Experience, and Perspective

Physicians attributed value to respecting the patient as a person, including their values and preferences, as a key precursor for connecting with patients about what is most important to them. For 1 physician, connection starts with an appreciation for the person. Other physicians said respecting patients involved working on what is important to them according to their values and preferences. Respect was additionally conveyed through communicating their acceptance of the patient regardless of the patient’s final decision.

### *Engaged Curiosity*: Curiosity for the Patient’s Story

The physicians interviewed had an underlying curiosity about their patients, which they described as important to fostering connection and sharing compassion. Curiosity manifested in several different ways. One physician described a genuine desire to understand what the patient’s expectations were for the clinical visit. Another physician had curiosity for the patient’s illness experience, and sought to understand what was important to the patient when they struggled to articulate it. Curiosity also played a role when 1 physician shared his attempt to understand the emotions a patient was experiencing. This curiosity for patients’ goals, needs, and emotions enabled providers to ask questions and elicit information that helped them understand where their patient was coming from and provide care in a genuine and meaningful way.

### *Focused Listening*: Value Listening With Concentration on What the Patient Is Saying

The physicians stated the importance of focusing on what the patient was trying to communicate by emphasizing the importance of trying to provide their undivided attention. Several described examples of how their mind organically wandered to their follow-up questions or their work-up, but stated their efforts to redirect their mind to focus on what the patient was saying. Another provider described turning away from her computer to face the patient, and focus solely on them, especially when they were sharing something vulnerable.

### *Mutual Participation*: Appreciation for the Patient’s Agency in Their Own Health and Healing

Physicians spoke about creating an interaction characterized by a balance of power between both participants as an important step toward improved connection. This was described as the physician serving as an expert in medicine while the patient is the expert in their own values, experience, and health goals. One physician stated his attempt to negotiate or compromise with patients, but declared that the patient should, nevertheless, have the final say. Another physician acknowledges a patient’s authority over their health by approaching their patient encounters with humility and remembrance of the patient’s survival thus far.

### *Self-Awareness*: Appreciation for One’s Own Values, Boundaries, and Emotions as They Arise in the Patient-Physician Relationship

When describing their interactions with patients, a number of interviewees shared ways they acknowledge themselves, their own emotional reactions and boundaries in the patient-physician relationship and how this awareness has enabled connection with patients. For instance, 1 provider said he has vocalized the disconnect he was feeling with a patient, and in naming it aloud, found it created an opportunity for the patient to express their true preferences and needs. Another provider similarly acknowledged that the conversation she was having with her patient was not going anywhere, which effectively served as a reset button to help remedy the situation. See Table 1 for an overview of themes and exemplary quotes. We present the themes here as principles that guide our physicians during their engagement with patients as opposed to prescriptive behaviors they follow.

**Table 1.** Theme Definitions and Illustrative Quotes From Physician Interviews.

Theme	Definition	Exemplary quotes
Respect for the patient	Appreciation for the patient as a whole person, including their beliefs, values, personal experience, and perspective	“Really appreciate who the person is.” “It’s fine if you decide to do something that I don’t recommend. That’s fine, I’m still going to care about you and care about your health.” “[Building trust] is showing commitment to [the patient] and respecting them as a person. Coming from where they are as much as possible.”
Engaged curiosity	Curiosity for the patient’s story	“What does this [illness] mean to you?” “If a patient is really reluctant to take a medication, [try] to explore that for a minute, or if they’re asking for some sort of test or evaluation that doesn’t seem appropriate, try to understand where that’s coming from and work with them to address whatever the underlying concern is.” “This is what I am hearing, is that correct? This is what I’m thinking about what you’re bringing up. What do you think about that? Does that resonate or not resonate with you?”
Focused listening	Value listening with concentration on what the patient is saying	“I’ve learned to just sit and listen and be present for when patients share their story about what’s going on with them, and what’s of interest to them, and really just giving them the space to talk about that and overcoming the urge to interrupt or direct the conversation as much as I might have say, ten years ago, earlier in my practice.” “[I] try not to immediately jump forward to what my next three questions are going to be or what test I am going to order, or who I am going to refer them to, but [I try to] be able to just listen.” “I really try to do the big things of making eye contact, and especially if somebody is expressing a really deep emotion. If they are crying, or telling about something really intimate, then I’ll stop, turn away from the computer, face them, mirror their body language.”
Mutual participation	Appreciation for the patient’s agency in their own health and healing	“You want to acknowledge that they’re the ones that are going to make all the decisions once you get clear what the clinical plan is.” “[Have] the resilience and the humbleness and respect that these folks are surviving without you.” “I don’t see myself as sitting there telling them what to do, but trying to engage them in what they’re interested in to improve their health.”
Self-awareness	Appreciation for one’s own values, boundaries, and emotions as they arise in the patient-physician relationship	“[Sometimes I will explicitly say] ‘I just don’t feel like we have a connection. I’m sorry, I wish we did’.” “[I will acknowledge] okay, this isn’t working. What’s going on? We’re not going anywhere.” “When I walk out of the room of that difficult encounter, I will sit at my desk for a moment before I jump into charting, or logging in, or doing whatever else needs to be done, just to be with that for a moment and to acknowledge that was really difficult, and not just try to blow past it and tough it out.”

## Discussion and Conclusions

### Discussion

In this study, we analyzed interviews of physicians with strong interpersonal communication skills to characterize their approach toward establishing connections with patients. Five themes emerged from the interviews: Respect for the patient, Engaged Curiosity, Focused Listening, Mutual Participation, and Self-Awareness. Below, we contextualize each of these themes within the existing landscape of care delivery models.

The *Respect for the Patient* that physicians describe was best captured by Carl Rogers, when he first emphasized the need for a provider to hold their client in high regard. He stated that this humanistic approach to therapy would both enable the patient to grow psychologically and reduce their suffering.^16^ Mary Catherine Beach, an expert in respect and relationships in healthcare, describes respect as going beyond the dominant “American” bioethical conception of respect for autonomy. She suggests the general public’s idea of respect is more in line with the European bioethical consensus statement that includes respect for not only autonomy but also dignity, integrity, and vulnerability.^17,18^ If a person perceives they are fundamentally valued in a relationship, it is understandable how they would feel more secure in sharing vulnerable information, especially when that information is emotionally charged. It is also often during these vulnerable conversations that connection with patients is felt. This is not to say that disclosure of vulnerable information is needed for connection, but what is needed is the perceived safety that otherwise enables one to disclose vulnerable information.

*Focused Listening* and *Engaged Curiosity* demonstrate a sustained interest in the patient’s perspective and their experience. The physicians described having curiosity about their patient’s life, goals, preferences, and illness experience and also attributed value to giving their undivided attention to gather this information. The care and time a physician takes to obtain and then confirm they have an accurate understanding of what their patient is saying demonstrates that the patient’s thoughts and experiences are of value. Other studies have similarly demonstrated that curiosity and subsequent focused listening enable trust building, increase understanding, and improve connection.^3,19^

For *Mutual Participation*, the physicians in this study described the importance of collaborating with patients and working alongside them on their care as opposed to telling them what to do. They also mentioned the importance of empowering the patient to be involved in managing their own care. This is similar to shared-decision making, which has been the more prominent approach to routine clinical practice.^20^ The primary difference is a particular emphasis on empowering the patient to take control over their health decisions as opposed to arriving at a collaborative agreement.

Multiple physicians in the study acknowledged their own personhood when considering their interactions with patients which suggests that they attribute value to acknowledging how the patient is affecting *them* and how *they* feel in the encounter with the patient. This *Self-Awareness* acknowledges the resilience, well-being, and emotional attentiveness that is required on the part of the physician when working on someone else’s health. Metacognition is important in the development of physicians’ clinical skills, including medical decision making. Our findings support assertions made in previous literature that metacognitive skills such as reflection and self-awareness are also important precursors to connecting with patients and minimizing burnout.^21,22^

### Innovation

**Figure 1.**
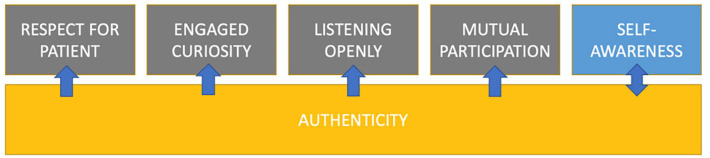
Conceptual map of emergent themes.

While similar physician communication skills as these have been identified in prior studies,^9^ a novel element that emerged from this analysis was a shared value of authenticity as a guiding principle underlying each of the 5 themes (Figure 1). In this work, authenticity was understood as a state of being, where one’s internal state mirrors the words and behaviors one externalizes. For instance, not simply acting and speaking in a respectful manner, but internally experiencing respect for the patient’s experience, decision, etc. regardless of whether one agrees. While never directly asked about authenticity, the interviewed physicians touched on the importance of authenticity through various descriptions of their approach to build connection, such as “the most fundamental way to build trust is being real with patients.” Another example was when one said they “try to be honest” followed by “sometimes I go to the extreme of just acknowledging [out loud] a disconnect.” The example this provider gave suggests their use of the word “honest” is not referring to full disclosure of medical information which has been found in the literature to build patient trust,^23^ but instead refers to the action of disclosing one’s internal experience with the intention of establishing connection or repairing a rupture. In this analysis, self-awareness was the 1 theme that did not have the same directional relationship as the others when considering authenticity. From the results of the interviews, one’s self-awareness is both a technique that is used to build connection with patients, but also serves to facilitate authentic connection. With the exception of only a few studies,^24-26^ examining physician self-awareness in the medical encounter is sparse. While metacognitive interventions centered on improving self-awareness can lead to habits that foster relationship-centered care,^21^ additional research on how to foster self-awareness in medical trainees would be helpful.

#### Set of behaviors or internal states?

The themes identified cannot be filed into a behavior or mindset category because they instead occupy both. The physicians in this study describe a mindset that manifests as behavior which highlights the importance of authenticity for building connection as opposed to a set of prescriptive behaviors. Mutual participation, or working alongside patients, will not work if internally the physician lacks a desire to collaborate equally with the patient; studies have shown that patients are accurate in perceiving how a physician feels about them.^27^ A current Behavioral Medicine textbook similarly stresses the need to tailor communication approaches to one’s own style, noting “if a clinician says something he does not believe, with the goal of manipulating the patient, it is likely to be detected and to backfire. We all have had the feeling of being patronized by service workers trained in customer satisfaction. However, if we express what we imagine the patient is feeling with the goal of strengthening a connection, the patient is likely to detect the authentic intent and forgive any awkwardness in the expression.”^28^ (p.21) Accordingly, this study encourages using communication techniques when the user is able to inhabit the words and actions they choose. This added, yet important, component will likely facilitate rapport and foster relationship-centered care.

Currently, there is very little in the medical literature describing how to conduct oneself authentically with patients, however the one recent study by Han et al^29^ that had expert physicians analyze “authentic resident communication skills” for communication competency yielded themes with significant overlap to ours. Similar themes included “authentic conversation by active listening and questioning,” “designing questions using both open-ended and closed questions,” “making agenda setting explicit to all parties at the beginning,” identifying preconditions of shared decision making (such as patient education and understanding patient contextual factors), and “multifaceted empathy demonstrated in multiple ways” (however “empathy” was not defined in this study). This convergence in communication behaviors perceived to facilitate, and be reflective of, authentic clinical engagement is encouraging, and points toward strategies for clinical communication training that may better facilitate both clinician well-being and improved patient care.

Broom et al^30^ studied authenticity in the caregiver experience while caring for a loved one with a terminal illness. In their study they describe authenticity in interpersonal relationships as the capacity to be “true” to one’s sense of self while in a relationship with another as opposed to displaying a socially expected behavior. They found that many caregivers experienced inauthenticity in their interpersonal relationships (e.g., hiding their exhaustion or resentment toward a dying loved one because of the social pressure to care selflessly for the dying family member) and that this led to a negative self-evaluation. The authenticity in this study focuses on the experience of the person who is acting inauthentically as opposed to how their inauthenticity is perceived by another, which was not discussed by the physicians in our study. However, it may be worthwhile to study the tension, and subsequent impact on physician wellness, that arises when physicians experience a misalignment between the way they genuinely feel toward a particular patient and the social and professional expectation of how to feel toward patients.

While authenticity and genuineness of character have been described as an important component of relationship centered care (RCC), concrete strategies for translating this value into behavior are still nascent. RCC, unlike patient-centered care, makes explicit that the clinician, along with the patient, is a unique individual with their own perspective and values and these also deserve acknowledgement in the patient-physician relationship.^10^ RCC emphasizes clinician authenticity in the sense that the physician must strive for congruency between their internal thoughts and feelings and what they are externalizing to the patient. Studies have shown that there is also a negative cost of inauthentic engagement for clinicians; projecting a professionally desired image instead of one’s experienced emotions is linked to increased psychological demands, burnout, and lower job satisfaction.^31-34^ Furthermore, both physicians and patients are highly accurate at perceiving how well liked they are by the other.^27^ Thus, acknowledging the authentic emotion and affinity present (or lack thereof, as our physicians described) is a more successful strategy for rapport building. While system-level change is undoubtedly needed to cultivate physician well-being, small rituals such as pausing and re-centering before a patient encounter have been identified as effective self-care strategies,^9^ which may better position a physician to present their authentic-self to patients.

Because the patient-physician relationship is a human social interaction, it is not realistic to suggest that a physician is capable of holding a high level of regard, curiosity, and interest in every patient. It is also important to acknowledge the energy involved in mentally regulating one’s internal and external state. However, we speculate that if a physician is able to employ behaviors that are both in line with communication techniques documented in prior literature, and confirmed in this study *and* feel true in the moment they use them, that this will allow for deeper patient-physician connection.

### Limitations

There are several biases inherent in this study. First, the study’s sample size is small and physicians were recruited by convenience sampling based on leadership and peer recommendation which may limit generalizability. Second, participants in the study described strategies that they use, however self-report is not always reliable and as a result may not capture actual behaviors and attitudes. Self-report may be additionally biased by recall problems and retrospective reconstructions of events. Third, these behaviors were extrapolated from interviews with outpatient internal medicine physicians and are not fully generalizable to other specialties or inpatient settings. An additional limitation is that, because we were analyzing previously collected data, we are unable to determine whether data saturation occurred, as most definitions of saturation rely on ascertaining the point at which new information is no longer emerging during data collection.^35^ As a result, it is possible that additional interviews with physicians known for their communication skills may yield additional insights. While the aforementioned limitations curtail our ability to generalize, study participants were selected specifically for their extraordinary skills in connecting with patients. This “positive deviance” approach allows us to identify practices associated with exceptional performance.^36^

## Conclusions

Physician authenticity in the patient-physician relationship is important for rapport building and guidance on achieving this quality is needed as medicine continues to adopt a relationship-centered approach. The 5 themes identified in this study are known to improve patient-physician relationships, however, utilizing these approaches with authenticity, where one’s external behavior is in accord with one’s internal state, is an important addition to communication techniques that strengthen rapport and connection in the patient-physician relationship.

### Practice Application

One of the most important ways to improve authentic connection with patients is to improve physicians’ capacity to understand, be sensitive to, and vicariously experience the feelings of a patient. This can be achieved by diversifying the physician workforce, not just in terms of racial identity, but by ensuring physicians come from a variety of socioeconomic backgrounds, religious affiliations, sexual orientations, and other minoritized experiences, as is evident from a growing body of literature showing a connection between greater diversity and improved physician-patient interactions.^37,38^ At present, even physicians and medical students with racial backgrounds traditionally underrepresented in medicine are disproportionally coming from high income families.^38,39^ However, previous studies have shown that discordance between physicians’ and patients’ socioeconomic status may negatively affect communication effectiveness and subsequent patient adherence and outcomes.^40,41^ Assembling a physician workforce that can better identify with the patients they work alongside will exponentially improve physicians’ ability to understand, connect, and even share similar experiences.

Second, when it comes to optimizing the patient-physician relationship, there is no standard approach to patient interactions, especially when the goal is authentic relationships. Authentic connection relies on both parties to honestly express their thoughts and feelings. To honor the professional standards in medical care, perhaps it would be worthwhile to highlight and reinforce concepts of emotional-regulation and boundary setting in medical education such that medical students learn how to tactfully and mindfully share their own thoughts and feelings with the patient (see Marroquín and Vine^42^ for evidence-based strategies). Additional research on the value of physician self-disclosure is needed; very few studies^25,43,44^ to date have examined its value, but preliminary evidence supports its usefulness and found no evidence of any training provided for physicians in how best to use self-disclosure as a clinical tool. By providing targeted training for physicians in how to engage authentically with patients, which should include training in ascertaining the appropriateness of self-disclosure as a means of finding common ground with patients, physicians will gain the skills necessary to better validate patients’ experience, demonstrate understanding, and also better protect their own well-being in routine clinical encounters.

Finally, a culture shift in medical education is needed to prioritize caring as well as emphasizing student well-being to avoid the cynicism reported as a result of medical training.^45^ Given the historical tension between competency and warmth or caring in medicine,^46^ significant effort should be made to incorporate non-cognitive attributes, including compassion and self-awareness, as critical competencies to be taught and assessed.^47^ While some may question if these attributes are modifiable, research suggests that such skills can be taught through intentional education, fostering positive learning environments, and modeling authenticity by faculty.^48^ We propose that that by synthesizing insights from physicians, such as those in the current study, who have found authentic ways to embody relationship-centered care, we can provide clinicians with relational tools for care delivery that protects the well-being of patients and physicians alike.

## Appendix A

### Provider and Staff Interviews

January 22, 2018

*The Stanford* Presence 5 *team is studying opportunities to enhance the clinical encounter. As part of this effort, we are gathering information about what clinicians do and say to connect with their patients, and strategies that they use to be fully present during clinical encounters and bring intentionality to their clinical care.*

1. What is your profession and title?
2. Can you tell me what you love about being a doctor?
  a. Was that why you went into medicine?
  b. How does your current work compare to what you’d expected?
3. For how many years have you been practicing medicine (include years post-residency, beginning with fellowship if applicable):___
4. How many patients do you see per week on average?
5. We are interested in learning about strategies that you use to connect with your patients. Are there specific things that you say or non-verbal gestures or actions that you use to help you engage with patients, build their trust, and encourage them to open up to you?

Prompt: “Can you give me an example of what that might sound/look like?”

6. Is there anything specific that you do to build trust [with patients and family or caregivers]?
7. Is there anything that you do to help you be more present (or fully emotionally available) during your interactions with patients?

Prompt: For example, is there anything that you do before, during or after interactions to facilitate your engagement in these interactions? Are there any rituals you have to center yourself and bring *intentionality* to what you are doing?

8. Is there anything in your environment that helps or hinders your ability to be present and connect with your patients?
9. How does technology help or hinder your ability to be present and connect with your patients?
  a. What other things hinder your ability to be present during a clinic visit?
10. What would you need to have happen in order for clinic visits to be fulfilling for you? (bare minimum for fulfillment, as well as how to make them more fulfilling)
11. What would need to change for you to be able to have your best clinic day more often?
12. Sometimes connecting deeply with a patient can lead to intense interactions. Is there anything you do to establish boundaries with patients in these instances?

Prompt: Boundaries to protect yourself psychologically and also to keep your visits within allotted time?

13. I am curious about how you know that you’ve made a connection with a patient. Can you think of a specific recent interaction and walk me through how you knew you made a connection?
14. Have you had times when it was difficult to connect with a patient? Why was that? What did you do?
15. What are the sociodemographics of the group you primarily working with (race/ethnicity, age, languages spoken, and insurance status)? Do you change your strategies when you are trying to connect with different types of patients? How so?
16. Do you change your strategies for connecting with patients in different situations (eg, in a crisis)?
17. Has your clinical style changed as you’ve gained more experience? If so, in what ways? [For example, are you more informal/formal/supportive/challenging/etc.]
18. What interpersonal skills do you now possess that you did not have earlier in your career? How did you develop those?
19. Who or what do you turn to for clinical guidance or resources when you could use additional support?
20. Does clinical work affect you or your family in different ways now than it once did earlier on in your practice? If so, what do you feel has contributed to these differences?
21. What advice would you give to medical students, residents and younger physicians entering your field?
22. What part of your work is most meaningful to you? What would allow you to do/experience more of this than you currently are?
23. What challenges have surprised you the most about your job? What do you find most frustrating?
24. What are the largest unmet patient needs that you’ve observed in your work?
25. What are the unmet provider needs that you’ve observed/experienced in your work? What do you think might improve/address these?
26. We want to know about what you tell your patients to help them get the most out of their clinic visits? What strategies, tools, patient education, apps do you share with them?

## References

[1] Peabody F. The care of the patient. JAMA. 1927;88(12):877-878. doi:10.1300/J013v36n01_07 4585472

[2] Haverfield MC Tierney A Schwartz R , et al. Can patient – provider interpersonal interventions achieve the quadruple aim of healthcare ? A systematic review. J Gen Intern Med. 2020;35(7):2107-2117. doi:10.1007/s11606-019-05525-2 31919725 PMC7351919

[bibr3-21501319231225996] Roter DL. Improving physicians’ interviewing skills and reducing patients’ emotional distress. Arch Intern Med. 1995;155:1877. 10.1001/archinte.1995.00430170071009 7677554

[4] Bodenheimer T Sinsky C. From triple to quadruple aim: care of the patient requires care of the provider. Ann Fam Med. 2014;12(6):573-576. doi:10.1370/afm.1713 25384822 PMC4226781

[5] Epstein RM Franks P Fiscella K , et al. Measuring patient-centered communication in patient-physician consultations: theoretical and practical issues. Soc Sci Med. 2005;61(7): 1516-1528. doi:10.1016/j.socscimed.2005.02.001 16005784

[6] Haskard Zolnierek KB Dimatteo MR. Physician communication and patient adherence to treatment: a meta-analysis. Med Care. 2009;47(8):826-834. doi:10.1097/MLR.0b013e31819a5acc 19584762 PMC2728700

[7] Frankel RM Stein T. Getting the most out of the clinical encounter: the four habits model. J Med Pr Manag. 2001;16: 184-191. 11317576

[8] Sullivan L. Doctor offers patient-communication PEARLS. Caring Ages. 2008;9:4.

[9] Zulman DM Haverfield MC Shaw JG , et al. Practices to foster physician presence and connection with patients in the clinical encounter. JAMA. 2020;323(1):70-81. doi:10.1001/jama.2019.19003 31910284

[10] Beach MC Inui T Frankel R , et al. Relationship-centered care: a constructive reframing. J Gen Intern Med. 2006;21:S3-S8. doi:10.1111/j.1525-1497.2006.00302.x PMC1484841 16405707

[11] Altamirano J Kline M Schwartz R Fassiotto M Maldonado Y Weimer-Elder B. The effect of a relationship-centered communication program on patient experience and provider wellness. Patient Educ Couns. 2022;5:1988-1995. doi:10.1016/j.pec.2021.10.025 34772532

[12] Garcia LC Shanafelt TD West CP , et al. Burnout, depression, career satisfaction, and work-life integration by physician race/ethnicity. JAMA Netw Open. 2020;3(8):1-13. doi:10.1001/jamanetworkopen.2020.12762 PMC7414389 32766802

[13] Schwartz R Haverfield MC Brown-Johnson C , et al. Transdisciplinary strategies for physician wellness: qualitative insights from diverse fields. J Gen Intern Med. 2019;34(7):1251-1257. doi:10.1007/s11606-019-04913-y 31037542 PMC6614234

[14] Brown-Johnson C Schwartz R Maitra A , et al. What is clinician presence? A qualitative interview study comparing physician and non-physician insights about practices of human connection. BMJ Open. 2019;9(11):1-8. doi:10.1136/bmjopen-2019-030831 PMC6858153 31685506

[15] Braun V Clarke V. Using thematic analysis in psychology. Qual Res Psychol. 2006;3(2):77-101.

[16] Rogers CR. Client-Centered Therapy: Its Current Practice, Implications, and Theory. Reprint. Constable & Robinson Ltd; 1995.

[17] Beach MC Branyon E Saha S. Diverse patient perspectives on respect in healthcare: a qualitative study. Patient Educ Couns. 2017;100(11):2076-2080. doi:doi:10.1016/j.pec.2017.05.010 28602565 PMC6400635

[18] Beach MC Roter DL Wang N-Y Duggan PS Cooper LA. Are physicians’ attitudes of respect accurately perceived by patients and associated with more positive communication behaviors? Patient Educ Couns. 2006;62(3):347-354. doi:10.1016/j.pec.2006.06.004 16859867 PMC3119350

[19] Kashdan TB Roberts JE. Trait and state curiosity in the genesis of intimacy: differentiation from related constructs. J Soc Clin Psychol. 2004;23(6):792-816. doi:10.1521/jscp.23.6.792.54800

[20] Elwyn G Frosch D Thomson R , et al. Shared decision making: a model for clinical practice. J Gen Intern Med. 2012;27:1361-1366. doi:10.1007/s11606-012-2077-6 22618581 PMC3445676

[21] Ko CJ Kim R Fortin AH Spak JM Hafler JP. Relationship-centered care in the physician-patient interaction: improving your understanding of metacognitive interventions. Cutis. 2021;107(6):320-324. doi:10.12788/cutis.0266 34314316

[22] Iskander M. Burnout, cognitive overload, and metacognition in medicine. Med Sci Educ. 2019;29(1):325-328. doi:10.1007/s40670-018-00654-5 34457483 PMC8368405

[23] Hillen MA de Haes HCJM Smets EMA . Cancer patients’ trust in their physician-a review. Psychooncology. 2011;20:227-241. doi:10.1002/pon.1745 20878840

[24] Buetow S Elwyn G. The window-mirror: a new model of the patient- physician relationship. Open Med. 2008;2(1):e20-e25. PMC3091589 21602948

[bibr25-21501319231225996] Beckman HB Wendland M Mooney C , et al. The impact of a program in mindful communication on primary care physicians. Acad Med. 2012;87(6):815-819. doi:10.1097/ACM.0b013e318253d3b2 22534599

[26] Longhurst M. Physician self-awareness: the neglected insight. CMAJ. 1988;139(2):121-124. 3390780 PMC1268026

[27] Hall JA Horgan TG Stein TS Roter DL. Liking in the physician-patient relationship. Patient Educ Couns. 2002;48(1): 69-77. doi:10.1016/S0738-3991(02)00071-X 12220752

[28] Fortin A VI . Empathy. In: Feldman M Christensen J Satterfield J Laponis R , eds. Behavioral Medicine: A Guide for Clinical Practice. 5th ed. McGraw HIll; 2019:14-23.

[29] Han H Hingle ST Koschmann T Papireddy MR Ferguson J. Analyzing expert criteria for authentic resident communication skills. Teach Learn Med. 2022;34(1):33-42. doi:10.1080/10401334.2021.1977134 34542388

[30] Broom A Parker RB Kenny K. Authenticity, ambivalence and recognition in caring at the end of life and beyond. Soc Sci Med. 2019;239:112554. doi:10.1016/j.socscimed.2019.112554 31542650

[31] Psilopanagioti A Anagnostopoulos F Mourtou E Niakas D. Emotional intelligence, emotional labor, and job satisfaction among physicians in Greece. BMC Health Serv Res. 2012; 12(1):463. doi:10.1186/1472-6963-12-463 23244390 PMC3541956

[bibr32-21501319231225996] Jeung DY Kim C Chang SJ. Emotional labor and burnout: a review of the literature. Yonsei Med J. 2018;59(2):187-193. doi:10.3349/ymj.2018.59.2.187 29436185 PMC5823819

[bibr33-21501319231225996] Larson EB Yao X. Clinical empathy as emotional labor in the patient-physician relationship. J Am Med Assoc. 2005;293(9):1100-1106. doi:10.1001/jama.293.9.1100 15741532

[34] Brotheridge CM Grandey AA . Emotional labor and burnout: comparing two perspectives of “people work.” J Vocat Behav. 2002;60(1):17-39. doi:10.1006/jvbe.2001.1815

[35] Saunders B Sim J Kingstone T , et al. Saturation in qualitative research: exploring its conceptualization and operationalization. Qual Quant. 2018;52(4):1893-1907. doi:10.1007/s11135-017-0574-8 29937585 PMC5993836

[36] Bradley EH Curry LA Ramanadhan S Rowe L Nembhard IM Krumholz HM. Research in action: using positive deviance to improve quality of health care. Implement Sci. 2009; 4(1):1-11. doi:10.1186/1748-5908-4-25 19426507 PMC2690576

[37] Jetty A Jabbarpour Y Pollack J Huerto R Woo S Petterson S. Patient-physician racial concordance associated with improved healthcare use and lower healthcare expenditures in minority populations. J Racial Ethn Heal Disparities. 2022; 9(1):68-81. doi:10.1007/s40615-020-00930-4 33403653

[38] Schoenthaler A Ravenell J. Understanding the patient experience through the lenses of racial/ethnic and gender patient-physician concordance. JAMA Netw Open. 2020;3(11):e2025349. doi:10.1111/sipr.12029 PMC11478220 33165607

[39] Shahriar AA Puram VV Miller JM , et al. Socioeconomic diversity of the matriculating US medical student body by race, ethnicity, and sex, 2017 - 2019. JAMA Netw Open. 2022;5(3):2017-2019. doi:10.1001/jamanetworkopen.2022.2621 PMC8924719 35289863

[40] Franks P Fiscella K Beckett L Zwanziger J Mooney C Gorthy S. Effects of patient and physician practice socioeconomic status on the health care of privately insured managed care patients. Med Care. 2003;41(7):842-852. doi:10.1097/00005650-200307000-00008 12835608

[41] Epstein AM Taylor WC Seage GR. Effects of patients’ socioeconomic status and physicians’ training and practice on patient-doctor communication. Am J Med. 1985;78(1):101-106. doi:10.1016/0002-9343(85)90469-3 3966475

[42] Marroquín B Vine V. Emotion regulation in patients, providers, and the clinical relationship. In: Schwartz R Hall JA Osterberg LG , eds. Emotion in the Clinical Encounter. McGraw Hill; 2021. https://accessmedicine.mhmedical.com/content.aspx?bookid=3088&sectionid=257489201. Accessed October 21, 2023.

[43] Arroll B Allen ECF . To self-disclose or not self-disclose? A systematic review of clinical self-disclosure in primary care. Br J Gen Pract. 2015;65(638):e609-e616. doi:10.3399/bjgp15X686533 PMC4540401 26324498

[44] Lussier MT Richard C. Self-disclosure during medical encounters. Can Fam Physician. 2007;53(3):421-422. 17872674 PMC1949073

[45] Underman K Hirshfield LE. Detached concern? Emotional socialization in twenty-first century medical education. Soc Sci Med. 2016;160:94-101. doi:10.1016/j.socscimed.2016.05.027 27227696

[46] Howe LC Leibowitz KA Crum AJ. When your doctor “Gets it” and “Gets you”: the critical role of competence and warmth in the patient-provider interaction. Front Psychiatry. 2019;10:1-22. doi:10.3389/fpsyt.2019.00475 31333518 PMC6619399

[47] Warm EJ Kinnear B Lance S Schauer DP Brenner J. What behaviors define a good physician? Assessing and communicating about noncognitive skills. Acad Med. 2022;97(2): 193-199. doi:10.1097/ACM.0000000000004215 34166233

[48] Batt-Rawden SA Chisolm MS Anton B Flickinger TE. Teaching empathy to medical students: an updated, systematic review. Acad Med. 2013;88(8):1171-1177. doi:10.1097/ACM.0b013e318299f3e3 23807099

